# Efficacy of a Combination of N-Palmitoylethanolamide, Beta-Caryophyllene, Carnosic Acid, and Myrrh Extract on Chronic Neuropathic Pain: A Preclinical Study

**DOI:** 10.3389/fphar.2019.00711

**Published:** 2019-06-27

**Authors:** Yannick Fotio, Amina Aboufares El Alaoui, Anna Maria Borruto, Samantha Acciarini, Antonio Giordano, Roberto Ciccocioppo

**Affiliations:** ^1^School of Pharmacy, Pharmacology Unit, University of Camerino, Camerino, Italy; ^2^Section of Neuroscience and Cell Biology, Department of Experimental and Clinical Medicine, Universita’ Politecnica delle Marche, Ancona, Italy

**Keywords:** neuropathic pain, nutraceutical, PEA, beta-caryophyllene, carnosic acid, myrrh, gabapentin

## Abstract

Neuropathic pain (NP) is a common public health problem that poses a major challenge to basic scientists and health-care providers. NP is a complex problem with an unclear etiology and an often-inadequate response to current medications. Despite the high number of drugs available, their limited pharmacological efficacy and side effects hamper their chronic use. Thus, the search for novel treatments is a priority. In addition to pharmaceuticals, natural extracts and food supplements are often used to help treating patients with NP. One such supplement is Noxiall^®^, a commercially available combination of N-Palmitoylethanolamide (PEA), beta-caryophyllene; carnosic acid and myrrh. Here, we compare the efficacy of Noxiall^®^ to that of the medications gabapentin and pregabalin in the NP model of chronic constriction injury (CCI) using sciatic nerve ligation in mouse. Following CCI, mice developed a significant increase in mechanical allodynia and thermal hyperalgesia. Results showed that administration of either Noxiall^®^, pregabalin, or gabapentin significantly attenuated mechanical allodynia. The magnitude of the Noxiall^®^ effect was comparable to that of gabapentin or pregabalin. In addition, co-administration of non-effective doses of pregabalin and Noxiall^®^ resulted in a significant decrease in NP, suggesting an additive efficacy. Noxiall^®^ was efficacious also in reducing CCI-induced thermal hyperalgesia. These findings support the rationale of using natural remedies in conjunction with classical pharmacological agents to treat chronic NP.

## Introduction

Neuropathic pain (NP) is a debilitating public health problem that affects 7% to 8% of the global population (Bouhassira et al., 2008; Smith and Torrance, 2012). Reduced productivity, increased compensation costs, and the treatment of conditions related to neuropathic pain contribute to the substantial financial burden in disease management. NP often arises from a lesion or diseases of the somatosensory system (Dworkin et al., 2003; Costigan et al., 2009; Amin and Hosseinzadeh, 2012) and is considered a separate clinical entity regardless of the underlying aetiology (Attal et al., 2008). The pathophysiology of NP is complex and not fully elucidated. Animal studies have shown that various cell-mediated mechanisms, are associated with this condition (Taylor, 2009). For instance, pro-inflammatory and pro-nociceptive mediators are released from injured nerve fibers and adjacent immune cells (e.g., macrophages, astrocyte, and microglia) (Ji and Strichartz, 2004; Abbadie, 2005; Costigan et al., 2009), leading to maladaptive neuronal adaptations resulting in hyperalgesia, allodynia usually observed in chronic pain (Costigan et al., 2009; Taylor, 2009).

Drugs approved for NP treatment primarily reduce the transmission and transduction of nociceptive signals, but their efficacy is limited. Several meta-analysis and reviews on NP indicate that only a minority of patients with NP experienced 30% to 50% reduction in pain (Attal et al., 2006; Attal et al., 2010; Baron et al., 2010; Cruccu et al., 2010; Dworkin et al., 2010; Schmader et al., 2010). Epidemiological surveys also revealed that currently used drugs (tricyclic antidepressants, selective serotonin re-uptake inhibitors, pregabalin, gabapentin, lidocaine patches, capsaicin high-concentration patches, and opioids) have significant side effects (especially in elderly patients) that hamper their therapeutic use (Dworkin et al., 2007; Attal et al., 2011). Despite development of newer drugs and the increased use of polytherapies, the clinical outcome remains generally modest with the number need to treat number needed to treat [(NNT); the average number of patients who need to be treated to obtain one positive responder] to obtain 50% pain relief has been estimated to be 7 in the most positive clinical trials (Finnerup et al., 2010a; Finnerup et al., 2010b). Novel, more efficacious, and safer analgesics for long-term treatments are highly needed.

Recently, the use of phytoderivatives and nutraceuticals has gained growing interest in the field of pain treatment. The use of these remedies is proposed for both the management of pain and for the control of the side effects associated with analgesic drugs (i.e., phytoderivatives are often used to attenuate opioid-induced constipation). Among these remedies, N-Palmitoylethanolamide (PEA) has been broadly studied (Gabrielsson et al., 2016; Artukoglu et al., 2017; Gabrielsson et al., 2017; Guida et al., 2017; Passavanti et al., 2017).

PEA is a fatty acid belonging to the N-acetyl ethanolamides family. It has anti-inflammatory, analgesic, and neuroprotective properties (Esposito et al., 2012). Several animal studies and clinical trials have been conducted to assess the clinical relevance of PEA as a stand-alone analgesic agent or as a part of a polytherapy (Gatti et al., 2012; Hesselink and Hekker, 2012; Sasso et al., 2013; Gabrielsson et al., 2016). Based on the affinity of PEA (EC_50_: 3μM)for the peroxisome proliferator-activated receptor-α (PPARα) (Lo Verme et al., 2005), it is possible that its analgesic effects are, at least in part, because of its agonism at this nuclear receptor, which is known to have an important role in pain relief (Calignano et al., 1998; Lo Verme et al., 2005). In addition, PEA plays an important role in suppressing the inflammation by reducing the activity of the pro-inflammatory enzymes such as cyclooxygenase (COX), endothelial Nitric Oxide Synthase (eNOS), and inducible nitric oxide synthase (iNOS) (Costa et al., 2002) and by reducing immune cells activation (Calignano et al., 1998; Calignano et al., 2001).

Another natural product with powerful analgesic properties is myrrh, an aromatic rubber-resin extracted from a tree *Commiphora myrrha* (Dolara et al., 1996). Earlier studies have shown that of its sesquiterpenes, curzarene, and furaneudesma-1,3-diene, are largely responsible for the analgesic effect of myrrh extracts. The analgesic effect of these sesquiterpenes is prevented by treatment with the opioid antagonist naloxone, suggesting a mechanism of action mediated by opioid receptors (Dolara et al., 1996).

Other natural products have also been shown to offer potential benefits in the treatment of pain and chronic inflammation; among these beta-caryophyllene (BCP) and carnosic acid (Klauke et al., 2014; Alberti et al., 2017; Segat et al., 2017). BCP is a natural bicyclic sesquiterpene constituent of many essential oils, especially from *Cannabis sativa* (Gertsch et al., 2008), *Syzygium aromaticum* (Ghelardini et al., 2001), *Rosmarinus officinalis (Cinnamomun)* (Gertsch et al., 2008; Ormeno et al., 2008), and *Piper nigrum* (Jirovetz et al., 2002). BCP acts as an agonist at the CB2 receptor, a receptor principally expressed in immune cells, and the activation of which induces anti-inflammatory effects and reduces microglia activation (Howlett et al., 2002; Klauke et al., 2014). Carnosic acid, present in *Rosmarinus officinalis* and *Salvia officinalis*, shows marked anti-oxidant properties, protects cells from oxidative stress, and increases the expression of antioxidant enzymes (Satoh et al., 2008; Ibarra et al., 2011). Carnosic acid’s anti-inflammatory properties also include inhibition of cyclooxygenase 2 (Cox 2), reduced expression of both interleukin 1 beta (Il-1β) and tumor necrosis factor alpha (TNFα) and attenuation of leukocytes infiltration into the damaged tissues (Maione et al., 2017).

Recently these natural products were combined in a single formulation (Noxiall^®^) that has been marketed as a food supplement (Italian dietary supplement registry-code 88326). Considering the potential analgesic and anti-inflammatory efficacy of this mixture, we sought to determine its efficacy in animal models of chronic NP. For this purpose, using the classical chronic constriction injury (CCI) model by ligation of the sciatic nerve in mouse, we tested Noxiall^®^ on mechanical allodynia. Pregabalin and gabapentin, two antiepileptics approved as first-line treatment of NP, were used as comparators. We then evaluated the analgesic effect of Noxiall^®^ on CCI induced thermal hyperalgesia. Finally, we tested whether combination of Noxiall^®^ with pregabalin, would offer advantages over the antiepileptic alone.

## Materials and Methods

### Animals

Male CD1 mice (Harlan, Varese, Italy) weighing approximately 28 to 32 g at the beginning of the experiments were used (*n*_total_ = 188). They were fed with a standard laboratory diet and tap water *ad libitum* (4RF18, Mucedola, Settimo Milanese, Italy). Animals were kept at 23 ± 1°C with a 12-h light/dark cycle, light at 7:30 a.m. All animal manipulations were carried out according to the *European Community guidelines for animal care*. Formal approval to conduct the experiments described was obtained from the Italian Ministry of Health and the Organism Responsible for Animal Welfare of the University of Camerino (protocol no 1D580.5). All efforts were made to minimize animal suffering, and none of these mice were used in multiple studies. Few mice (*n* = 9) showed excessive discomfort reaching human endpoint. For ethical reasons, these animals were euthanized and removed from the study.

### Induction of Neuropathic Pain by Chronic Constriction Injury (CCI)

Neuropathic pain was induced according to the procedure described in earlier studies (Bennett and Xie, 1988). Briefly, mice were anesthetized by inhalation of a mixture of isoflurane and oxygen mixture. Under aseptic conditions, the right (ipsilateral) common sciatic nerve was exposed at the level of the middle thigh by blunt dissection. Proximal to the trifurcation, the nerve was carefully cleaned from the surrounding connective tissue, and three chromic cat gut ligatures (4-0, Ethicon, Norderstedt, Germany) were tied loosely around the nerve with about 1 mm between ligatures. The CCI model of mononeuropathy elicits a pain syndrome, that begins about 3 days after the nerve injury and reaches a plateau lasting between 7 and 30 days (Di Cesare Mannelli et al., 2014). In our experiments behavioral measurements were performed on days 4, 7, and 14.

### Treatments

Noxiall^®^ (FB-Health) tablets, a nutraceutical composition of PEA 600 mg; myrrh 50 mg, BCP 10 mg, and *Rosmarinus officinalis* 30.8 mg (20 mg in carnosic acid) were crushed with mortar and pestle to obtain a thin powder that was then suspended in a solution made of 95% of distilled water, and 5% of Tween 80. The final volume was calculated to achieve the following concentrations: PEA (15 mg/ml), Myrrh extract (1.25 mg/ml), BCP (0.25 mg/ml), and carnosic acid (0.5 mg/ml). These concentrations were obtained by suspending each tablet of Noxiall^®^ in 40 ml of vehicle (PEA 600 mg/40 ml = 15 mg/ml; Myrrh extract 50 mg/40 ml = 1.25 mg/ml; BCP 10 mg/40 ml = 0.25 mg/ml; carnosic acid 20 mg/40 ml = 0.5 mg/ml). Gabapentin (100 mg, Teva Pharma, Italy) pills were crushed in a mortar and suspended in a vehicle (95% of distilled water and 5% of Tween 80) to obtain a final concentration of 5 mg/ml. Specifically, each pill of gabapentin was suspended in 20 ml (100 mg/20 ml = 5 mg/ml) of vehicle. Pregabalin (LYRICA^®^ 150 mg, Pfizer, Italy) pills were also powdered and then suspended in a vehicle (95% of distilled water and 5% of Tween 80) to obtain a final concentration of 5 mg/ml (150 mg/30 ml = 5 mg/ml). These suspensions were administered orally twice a day (at 09:00 and 17:00) at 10 ml/kg of body weight. To obtain lower concentrations, dilutions were made from these stock solutions. To evaluate the effect of Noxiall^®^ on CCI-induced mechanical allodynia mice were treated for 14 days, starting 24 h after surgery. For the tail immersion test, mice were treated twice (at 09:00 and 17:00) and the test was conducted at 18:00. Using the same treatment schedules control mice were treated with vehicle.

The quality control of the active ingredients contained in Noxiall^®^ is reported in the technical spreadsheet reported as supplementary material (see **Supplementary Material** files). Briefly, specific compositions were: PEA (FrauPharma) 99% purity [high-performance liquid chromatography (HPLC)]. Myrrh (Biosfered, Italy; total furanodieni ≥ 40 g/Kg of which curzerene ≥20%, furanoeudesma-1,3-diene ≥30%, Lindestrene ≥8%, other furanodieni ≥5%. BCP from *Piper nigrum* (Biosfered); 80% purity, α-cariofillene 2%, other therpens (α-Pinene, β-Pinene, D,L-Limonene, α-copaene). Analysis carried out gas chromatography–mass spectrometry (GC-MS)/FID. *Rosmarinus officinalis* in 65% carnosic acid (Nutrafur, Spain, HPLC). Total diterpenes (%), 68.0 to 75.0; carbohydrates (%), 0.5 to 1.0; lipids (%), 20.0 to 25.0; proteins (Nx 6.25) (%), 0.5 to 1.0; other polyphenols, 0.5 to 2.0, mineral salts (%), 0.1 to 0.5.

### Von Frey Test to Measure Mechanical Allodynia

Animals were placed in 10 ×10 cm Plexiglas boxes equipped with a metallic mesh floor, 20 cm above the bench. Fifteen minutes of habituation was allowed before the test session. An autonomic Von Frey hair unit (Ugo Basile, Varese, Italy) was used according to previously described methods (Barrot, 2012; Martinov et al., 2013). Briefly, a gradually increasing pressure was applied to filaments through the mesh floor perpendicularly to the plantar surface of ligated and non-ligated (contralateral) hind paw: the withdrawal threshold was evaluated by applying forces ranging from 0 to 5 g. The paw sensitivity threshold was set as the minimum force required to induce a robust and immediate withdrawal of the paw. Three nociceptive threshold values were recorded at 15-s intervals and averaged. Measurements were performed 60 min after the 09:00 drug administration.

### Hot Plate Test to Measure Thermal Hyperalgesia

Animals (27) were gently dropped in the hot plate apparatus (Socrel DS37) with a heating platform (25 × 25 cm) made of aluminium, and a transparent cylindrical chamber (20 × 26 cm) made of Plexiglas. The platform was maintained at 48.0°C ± 1.0°C (Espejo and Mir, 1993; Baliki et al., 2005). Briefly, paw withdrawal latency was calculated using a timer that was started when the animal was released onto the preheated platform and stopped at the moment of withdrawal, shaking, or licking of either hind paw. Three paw withdrawal measures (separated by 2 min) were recorded and averaged. A 30-s cutoff time was applied to avoid tissue damages. Higher is the latency, lower is the pain perception.

### Open Field Maze to Measure Locomotor Activity

*N* = 21 mice were placed in an automated open field boxes (Med Associate, St. Albans, VT, USA) to quantify the locomotor activity of animals following treatment with Noxiall^®^ in comparison to gabapentin. Each animal was placed in the activity box, a square plastic box measuring 43 × 43 × 30 cm, and spontaneous locomotor activity parameters were monitored as described here (Seibenhener and Wooten, 2015). Activity was recorded for 10 min after placing the animal in the test cage. Locomotor activity of each mouse was automatically recorded by interruption of light beams, which were connected to an automatic software. The behavioral parameter observed was locomotion (as reflected by the travel distance). Between animals, the apparatus was cleaned with alcohol (70%) and dried with a clean cloth.

### Statistical Analysis

The effect of treatment on the paw withdrawal threshold over time was analyzed by “Two-Way ANOVA with “treatment” as between factor and “time” as within factor. Withdrawal threshold of the ipsilateral (CCI paw) and the contralateral (control) paws were analyzed by “One-Way” between factor (treatment) ANOVA. Response to the Von Frey test at 4, 7, and 14 days were further analyzed by separate ANOVAs. Similar analysis was used to evaluated the effect of drugs on thermal hyperalgesia Effect of treatment on locomotor activity was analyzed by “one-way” between factor (treatment) ANOVA. When appropriate, the Newman–Keuls multiple comparison test was used for *post hoc* analysis. Statistical significance was set at *p* < 0.05. Data represent the mean (±SEM) of 9–10 mice/group.

## Results

### Experiment 1: Effect of Noxiall^®^ and Gabapentin on CCI-Induced Mechanical Allodynia

**Figure 1:**
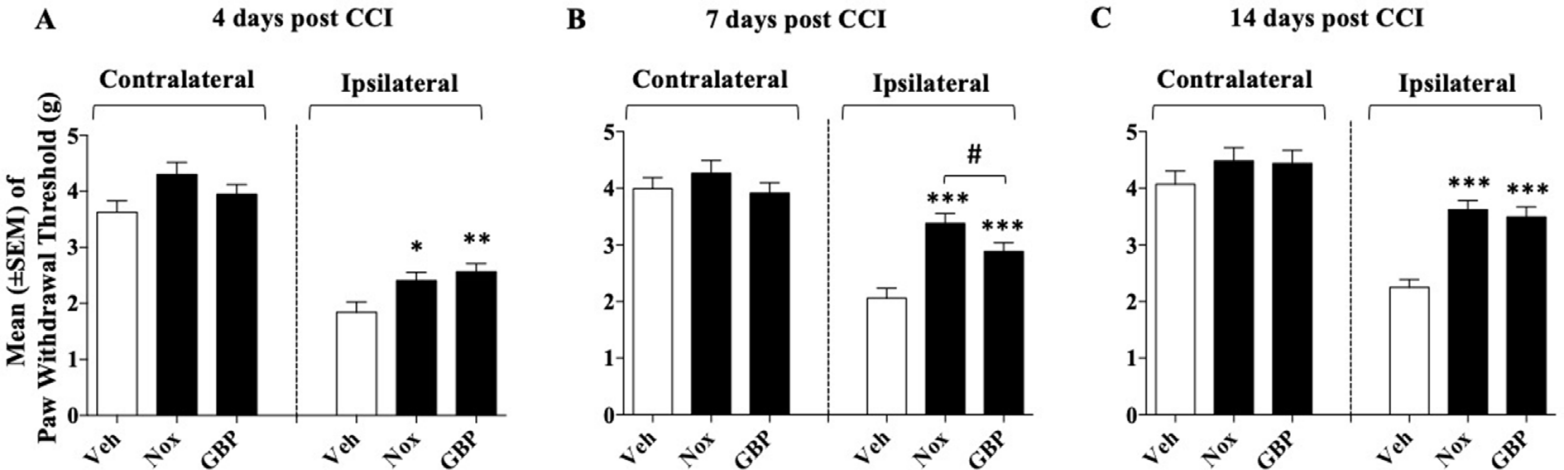
Effect of oral treatment with Noxiall® (Nox) and gabapentin (GBP) on chronic constriction injury (CCI)-induced mechanical allodynia in mice. Animals were treated twice a day with Noxiall® (PEA 300 mg/10 ml/kg, Myrrh 25 mg/10 ml/kg, beta-caryophyllene (BCP) 5 mg/10 ml/kg and *Rosmarinus officinalis* 6.16 mg/10 ml/kg), gabapentin (100 mg/10 mg/kg) or vehicle on mechanical allodynia: **(A)** at day 4; **(B)** day 7 and **(C)** day 14, respectively. Values represent the mean ± SEM. Difference from vehicle (Veh): *p < 0.05, **p < 0.01, ***p < 0.001, and ^#^p < 0.05 Nox vs GBP.

Mice were divided into three groups (*n* = 8–9/group at the beginning of the experiment): group 1, Noxiall^®^ (PEA 150 mg/10 ml/kg, Myrrh 12.5 mg/10 ml/kg, BCP 2.5 mg/10 ml/kg, and carnosic acid 5.0 mg/10 ml/kg); group 2, gabapentin (50 mg/10 ml/kg); group 3, vehicle (95% of distilled water and 5% of Tween 80/10 ml/kg). Drug treatments began 24 h after CCI surgery and continued until day 14. On days 4, 7, and 14 after sciatic nerve ligation the Von Frey test was carried out. *N* = 26 mice were used at the beginning of these experiments but 7 days following CCI surgery, one mouse of the vehicle group was excluded because it reached the human end-point. Thus, at days 4, 7, and 14, the number of mice used was 26, 25, and 25, respectively. As shown in **Figure 1**, in the control group the withdrawal threshold of the CCI paw with respect to the control paw remained markedly lower during the whole experimental period suggesting a stable allodynia following surgery. A two-way (factors: treatment and time) overall ANOVA revealed a significant effect of treatment [*F*_(2; 22)_ = 18.27; *p* < 0.0001], time [*F*_(2; 44)_ = 31.52; *p* < 0.0001] but not treatment × time interaction [*F*_(4; 44)_ = 1.586; *p* = 0.1949] on CCI. At the contralateral (control) paw overall ANOVA showed no effect of treatment [*F*_(2; 22)_ = 3.247; *p* = 0.0581], time [*F*_(2; 44)_ = 2.053; *p* = 0.14], and their interaction [*F*_(4; 44)_ = 0.2931; *p* = 0.8809]. To further evaluate the effect of treatment, single ANOVAs were used to analyze mechanical allodynia at the different timepoints. Results showed that treatments with either Noxiall^®^ or gabapentin significantly reduced the expression of mechanical allodynia at days 4 [*F*_(2; 23)_ = 7.4; *p* = 0.0033], 7 [*F*_(2; 22)_ = 33.69; *p* < 0.0001], and 14 [*F*_(2; 22)_ = 21.34; *p* < 0.0001]. The Newman–Keuls test revealed that both Noxiall^®^ and gabapentin significantly reduced the expression of mechanical allodynia throughout the treatment period. Treatments did not modify tactile reactions of control paw {day 4 [*F*_(2; 23)_ = 2.2603; *p* = 0.7731 (ns)]; day 7 [*F*_(2; 22)_ = 1.347; *p* = 0.2810 (ns)], and day 14 [*F*_(2; 22)_ = 0.9053; *p* > 0.05 (ns)]}.

### Experiment 2: Determination of the Dose Response Curve of Noxiall on CCI-Induced Mechanical Allodynia

**Figure 2:**
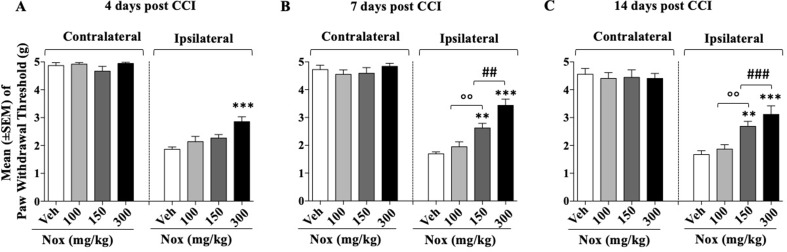
Dose–effect relationship of Noxiall^®^ (Nox) on CCI-induced mechanical allodynia at: **(A)** day 4 **(B)**; day 7; **(C)** day 14. Animals were treated twice a day with Noxiall^®^ at a high (PEA 300 mg/10 ml/kg, Myrrh 25 mg/10 ml/kg, BCP 5 mg/10 ml/kg, and *Rosmarinus officinalis* 6.16 mg/10 ml/kg); intermediate (PEA 150 mg/10 ml/kg, Myrrh 12.5 mg/10 ml/kg, BCP 2.5 mg/10 ml/kg and *Rosmarinus officinalis* 3.08 mg/10 ml/kg) low (PEA 100 mg/10 ml/kg, Myrrh 8.32 mg/10 ml/kg, BCP 1.66 mg/10 ml/kg, and *Rosmarinus officinalis* 2.04 mg/10 ml/kg) dose or vehicle (Veh). Values represent the mean ± SEM. Difference from vehicle **p < 0.01, ***p < 0.001, ^##^ *p* < 0.01, ^###^ *p* < 0.001 (300 vs 150 mg/kg), and ^oo^ *p* < 0.01 (150 vs 100 mg/kg). Where not indicated, differences with the vehicle were not statistically significant.

After 24 h from CCI surgery four groups of mice (*n* = 10/group at the beginning of the experiment) received three different doses of Noxiall^®^ or vehicle. Specifically, twice a day, group 1 received Noxiall^®^ (PEA 150 mg/10 ml/kg, Myrrh 12.5 mg/10 ml/kg, BCP 2.5 mg/10 ml/kg, and carnosic acid 5.0 mg/10 ml/kg); group 2, Noxiall^®^ (PEA 75 mg/10 ml/kg, Myrrh 6.25 mg/10 ml/kg, BCP 1.25 mg/10 ml/kg, and carnosic acid 2.5 mg/10 ml/kg); group 3, Noxiall^®^ (PEA 50 mg/10 ml/kg, Myrrh 4.16 mg/10 ml/kg, BCP 0.83 mg/10 ml/kg, and carnosic acid 1.67 mg/10 ml/kg); group 4, vehicle. Treatments continued for 14 days. On days 4, 7, and 14 after sciatic nerve ligation, the Von Frey test was carried out. Forty mice were used at the beginning of these experiments but due to excessive discomfort two mice (one vehicle and one Noxiall^®^ lowest dose) were sacrificed before day 7. Whereas other two mice (one Noxiall^®^ lowest dose and one Noxiall^®^ intermediate dose) were sacrificed before test day 14. Thus, the number of mice tested at days 4, 7, and 14 were 40, 38, and 36, respectively. On CCI-induced mechanical allodynia, a two-way (factors: treatment and time) overall ANOVA showed a significant effect of treatment [*F*_(3; 34)_ = 29.85; *p* < 0.0001], but no effect of time [*F*_(2; 68)_ = 0.9660; *p* = 0.3858] or treatment × time interaction [*F*_(6; 68)_ = 1.939; *p* = 0.087]. At the contralateral (control) paw, overall ANOVA showed no effect of treatment [*F*_(3; 34)_ = 0.4804; *p* = 0.6981], no effect of time [*F*_(2; 68)_ = 6.379; *p* = 0.0929] and no treatment × time interaction [*F*_(6; 68)_ = 0.3017; *p* = 0.8753]. To further evaluate the effect of treatment, single ANOVAs were used to analyze mechanical allodynia at the different timepoints. As shown in **Figure 2A, B, and C** in the control group the withdrawal threshold of ligated paw remained markedly lower with respect to the contralateral paw suggesting stable expression of allodynia after sciatic nerve ligation. ANOVA revealed a significant effect of the treatment at all timepoints {day 4, [*F*_(3; 36)_ = 7.845; *p* = 0.0004]; day 7 [*F*_(3; 34)_ = 21.54; *p* < 0.0001]; day 14 [*F*_(3; 32)_ = 10.29; *p* < 0.001]}. As shown in **Figure 2A**, on day 4 the Newman–Keuls test, revealed significantly (*p* < 0.01) reduced mechanical allodynia following the highest dose of Noxiall^®^ compared to vehicle. On day 7 (**Figure 2B**) and on day 14 (**Figure 2C**), the treatment attenuated the expression of mechanical allodynia at the intermediate (*p* < 0.01) and at the highest dose (*p* < 0.001) compared to vehicles. Noxiall^®^ did not modify the paw withdrawal threshold of the contralateral paw at any of the timepoint tested.

### Experiment 3: Determination of the Dose Response Curve of Pregabalin on CCI-Induced Mechanical Allodynia

**Figure 3:**
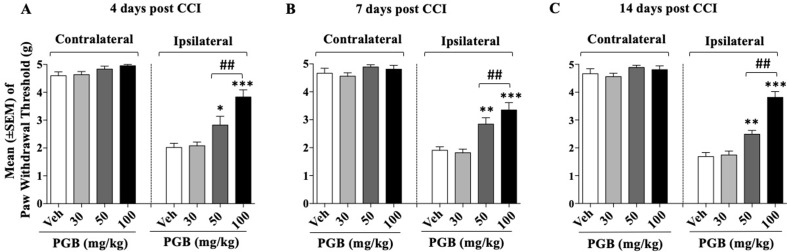
Dose–effect relationship of pregabalin (PGB) on CCI-induced mechanical allodynia at: **(A)** day 4 **(B)**; day 7; **(C)** day 14. Pregabalin (30, 50, or 100 mg/10 ml/kg) or vehicle were given twice a day. Values represent the mean ± SEM. Difference from vehicle: *p < 0.05, **p < 0.01, ***p < 0.001, and ^##^ *p* < 0.01 (100 vs 50 mg/kg). Where not indicated, differences with the vehicle were not statistically significant.

After 24 h from the CCI surgery, four groups of mice (*n* = 9–10/group) received different doses of pregabalin (15, 25, or 50 mg/10 ml/kg) or vehicle twice a day. Treatments continued for 14 days. On days 4, 7, and 14 after sciatic nerve ligation, the Von Frey test was performed. Thirty-eight mice were involved at the beginning of these experiments but due to excessive discomfort one mouse (pregabalin, 25 mg group) was sacrificed before test day 7. Other two mice (one vehicle and one pregabalin 15 mg) were sacrificed before test day 14. Thus, the number of mice tested at days 4, 7, and 14 were 38, 37, and 35, respectively. As shown in **Figure 3**, for the whole experimental period in the control group the withdrawal threshold of the experimental paw remained markedly lower respect to the contralateral paw suggesting stable expression of allodynia after sciatic nerve ligation. A two-way (factors: treatment and time) overall ANOVA revealed a significant effect of treatment [*F*_(3; 33)_ = 59.85; *p* < 0.0001], but not the effect of time [*F*_(2; 66)_ = 1.624; *p* = 0.2049] and treatment × time interaction [*F*_(6; 66)_ = 0.8566; *p* = 0.5315] on CCI. Overall ANOVA also showed no effect of treatment [*F*_(3; 33)_ = 2.426; *p* = 0.083], time [*F*_(2; 66)_ = 0.1780; *p* = 0.8374] and treatment × time interaction [*F*_(6; 66)_ = 0.3671; *p* = 0.8972] on the ipsilateral (CCI) paw. When single ANOVAs were used to further analyze CCI-induced mechanical allodynia at the different timepoints results revealed a significant effect of treatment at all timepoints {day 4, [*F*_(3; 34)_ = 14.59; *p* < 0.0001]; day 7 [*F*_(3, 33)_ = 14,46; *p* < 0.0001]; day 14 [*F*_(3; 31)_ = 38,19; *p* < 0.0001]}. As shown in **Figure 3A**, on day 4, the Newman–Keuls test, revealed significant reduction of mechanical allodynia at 25 mg/kg (*p* < 0.05) and 50 mg/kg (*p* < 0.01) of pregabalin compared with vehicle. On day 7 (**Figure 3B**) and on day 14 (**Figure 3C**) pregabalin markedly attenuated the expression of mechanical allodynia at 25 mg/kg (*p* < 0.01) and 50 mg/kg (*p* < 0.001) compared to vehicles. Drug treatment did not modify the paw withdrawal threshold of the contralateral paw at any of the timepoint tested.

### Experiment 4: Co-administration of Inactive Doses of Pregabalin and Noxiall^®^ on CCI-Induced Mechanical Allodynia

**Figure 4:**
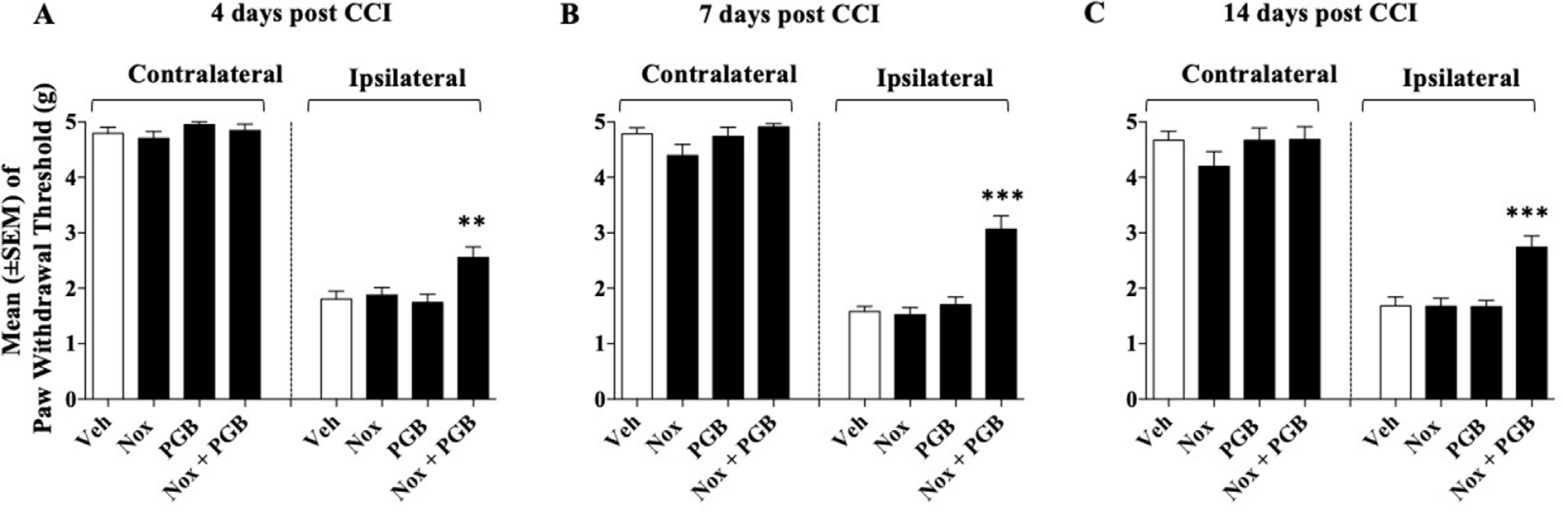
Effect of co-administration of an ineffective doses of Noxiall^®^ (Nox) and of pregabalin (PGB) on CCI-induced mechanical allodynia at: **(A)** day 4; **(B)** day 7; **(C)** day 14. Noxiall^®^ (PEA 100 mg/10 ml/kg, Myrrh 8.32 mg/10 ml/kg, BCP 1.66 mg/10 ml/kg and *Rosmarinus officinalis* 2.04 mg/10 ml/kg), pregabalin (30 mg/10 ml/kg) their combination (Nox + PGB) or vehicle (Veh) were given twice a day. Values represent the mean ± SEM. Difference from vehicle: **p < 0.01 and ***p < 0.001. Where not indicated, differences with the vehicle were not statistically significant.

Previous experiments showed that the lowest dose of Noxiall^®^ [PEA 50 mg/10 ml/kg, Myrrh 4.16 mg/10 ml/kg, BCP 0.83 mg/10 ml/kg, and carnosic acid 1.67 mg/10 ml/kg); and 15 mg/kg of pregabalin were both ineffective at reducing CCI-induced allodynia. Here, these ineffective doses were co-administered to evaluate the possibility of additive effects. After 24 h from the CCI surgery four groups of mice (*n* = 8-10/group) received either Noxiall^®^, pregabalin, the combination of both drugs or vehicle twice a day. Treatments continued for 14 days. *N* = 36 mice were involved at the beginning of these experiments but due to excessive discomfort one mouse (Noxiall^®^ group) was sacrificed before test day 7. Thus, the number of mice tested at days 4, 7, and 14 were 36, 35, and 35, respectively. On days 4, 7, and 14 after sciatic nerve ligation the Von Frey test was carried out. On CCI-induced allodynia a two-way (factors: treatment and time) overall ANOVA revealed a significant effect of treatment [*F*_(3; 32)_ = 34.93; *p* < 0.0001], but not the effect of time [*F*_(2; 64)_ = 0.1215; *p* = 0.8858] or treatment × time interaction [*F*_(6; 64)_ = 1.397; *p* = 0.2297]. At the contralateral (control) paw, overall ANOVA showed no significant effect of treatment [*F*(3; 32) = 1.096; *p* = 0.3709], no effect of time [*F*_(2; 64)_ = 2.695; *p* = 0.0754] and no treatment × time interaction [*F*_(6; 64)_ = 0.4040; *p* = 0.8737]. To further evaluate the effect of treatment, single ANOVAs were used to analyze mechanical allodynia at the different timepoints. As in previous experiments in the control group the paw withdrawal threshold of the CCI paw remained markedly lower with respect to the contralateral paw suggesting stable expression of allodynia after surgery. ANOVA revealed a significant effect of the treatment at all timepoints {day 4 [*F*_(3; 32)_ = 5,49; *p* = 0.0037]; day 7 [*F*_(3, 31)_ = 22.39; *p* < 0.0001]; day 14 [*F*_(3,31)_ = 10,24; *p* < 0.0001]}. On day 4 (**Figure 4A**), compared to vehicles the Newman–Keuls test revealed a significant reduction of mechanical allodynia only when Noxiall^®^ and pregabalin were combined (*p* < 0.01). As shown in **Figure 4B** and **Figure 4C** similar but more pronounced effects were detected on day 7 (*p* < 0.001) and day 14 (*p* < 0.001). Treatment with Noxiall^®^ or with Pregabalin alone did not exert significant effects compared to vehicles. Drug treatments did not modify the withdrawal threshold of the contralateral paw at any of the timepoint tested.

### Experiment 5: Effect of Noxiall^®^ and Gabapentin on CCI-Induced Thermal Hyperalgesia

**Figure 5:**
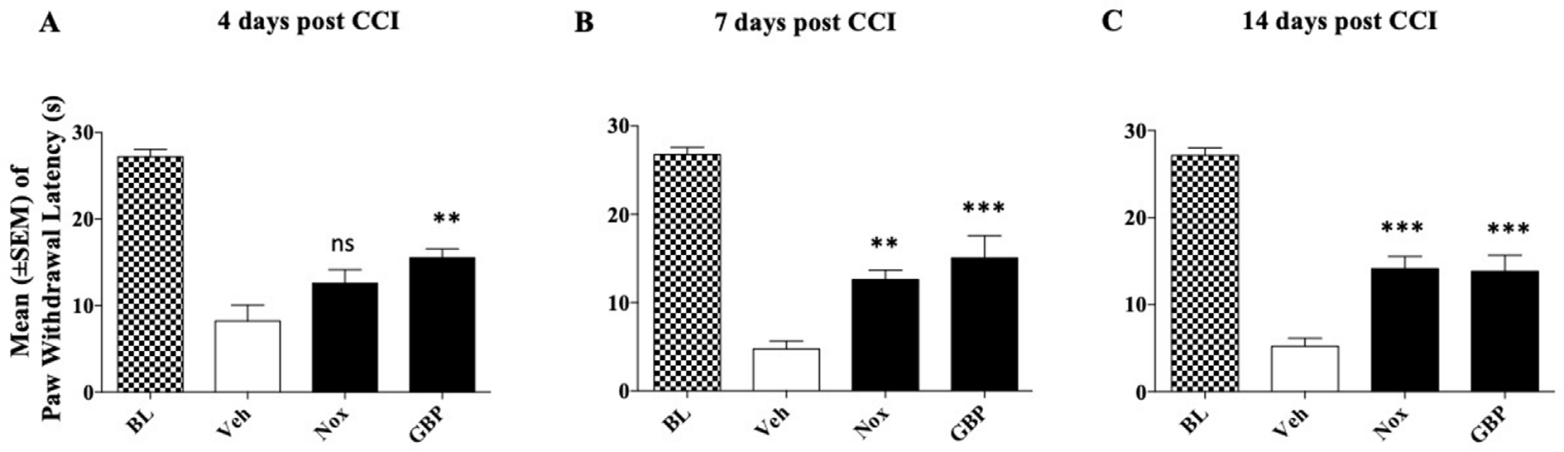
Effect of oral treatment with Noxiall^®^ (Nox) and GBP on CCI-induced heat hyperalgesia in male CD1 mice. Animals (*n* = 9/group) were treated twice a day with Noxiall^®^ (PEA 300 mg/10 ml/kg, Myrrh 25 mg/10 ml/kg, BCP 5 mg/10 mL/kg and *Rosmarinus officinalis* 6.16 mg/10 ml/kg), gabapentin (100 mg/10 mg/kg) or vehicle on mechanical allodynia: **(A)** at day 4; **(B)** day 7; and **(C)** day 14, respectively. Values represent the mean ± SEM. Difference from vehicle (Veh): **p < 0.01 and ***p < 0.001.

Mice were divided into three groups (*n* = 9/group at the beginning of the experiment) to be tested with the highest dose of Noxiall^®^ and gabapentin: group 1, received Noxiall^®^ (PEA 150 mg/10 ml/kg, Myrrh 12.5 mg/10 ml/kg, BCP 2.5 mg/10 ml/kg, and carnosic acid 5.0 mg/10 ml/kg); group 2 was treated with gabapentin (50 mg/10 ml/kg); group 3 was injected with vehicle (95% of distilled water and 5% of Tween 80/10 ml/kg). Treatments began 24 h after CCI surgery and continued until day 14. On days 4, 7, and 14 after sciatic nerve ligation the hot plate test was carried out. Twenty-six mice were used at the beginning of the experiment but 4 days following CCI surgery, 3 mice were excluded because they reached the human end-point. Thus 24 mice were included in the statistical analysis. As shown in **Figure 5**, in the control group the paw withdrawal latency of mice respect to their baseline values remained markedly lower during the whole experimental period suggesting a stable hyperalgesia following surgery. A two-way (factors: treatment and time) overall ANOVA revealed a significant effect of treatment [*F*_(2; 21)_ = 34.44; *p* < 0.0001], but not time [*F*_(2; 42)_ = 0.7612; *p* = 0.4734] and the interaction between factors [*F*_(4; 42)_ = 0.7052; *p* = 0.5923] on CCI. To further evaluate the effect of treatment, single ANOVAs’ were used to analyze heat hyperalgesia at the different timepoints. As reported in **Figure 5**, results showed that on day 4, a significant effect of treatments on thermal hyperalgesia was detected [*F*_(2; 21)_ = 5.806; *p* = 0.0095]. Newman–Keuls *post hoc* analysis revealed that treatment with gabapentin (*p* < 0.01) but not Noxiall^®^ significantly reduced the expression of heat hyperalgesia. Drug treatments showed a significant reduction in thermal hyperalgesia also at day 7 [*F*_(2; 21)_ = 10.43; *p* = 0.0007] and day 14 [*F*_(2; 21)_ = 12.46; *p* = 0.0003]. *Post hoc* tests showed a significant reduction in response to thermal stimuli with Noxiall^®^ at 7 (*p* < 0.01) and 14 days (*p* < 0.001) as well as with gabapentin (*p* < 0.001).

### Experiment 6: Effect of Noxiall^®^ and Gabapentin on Locomotor Activity

**Figure 6:**
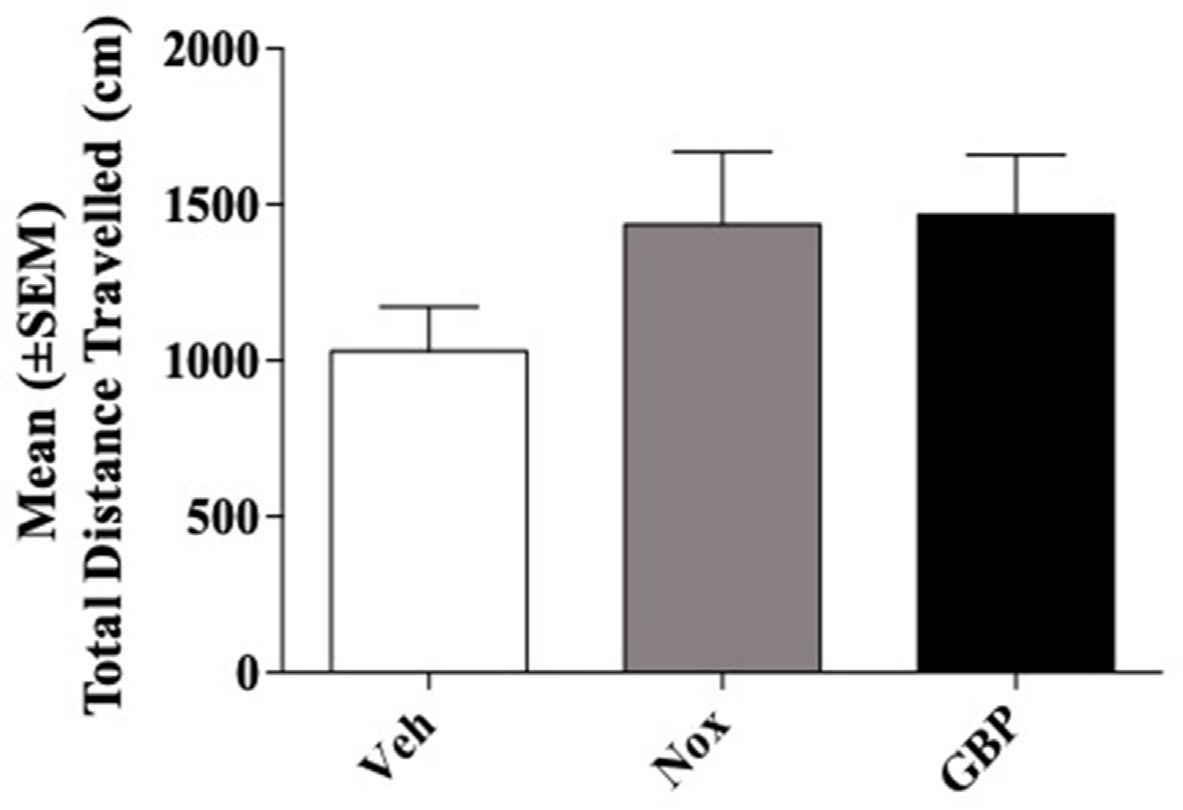
Total distance traveled in the open field test. Total distance in centimeters (cm) of their respective tracks were combined and statistically analyzed to visualize any differences in ambulation. With respect to the vehicle, mice treated with Noxiall^®^ or gabapentin did not show significant differences in the Open Field Maze (OFM) when total distance was measured. Data are expressed as the mean ± SEM.

CD1 mice (*n* = 21) were divided into three groups (*n* = 7/group). Group 1 received vehicle (95% of distilled water and 5% of Tween 80/10 ml/kg); group 2, the highest dose of Noxiall^®^ (PEA 150 mg/10 ml/kg, Myrrh 12.5 mg/10 ml/kg, BCP 2.5 mg/10 ml/kg and carnosic acid 5.0 mg/10 ml/kg); group 3, the highest dose of gabapentin (50 mg/10 ml/kg). Overall, ANOVA showed no effects of treatments on total distance travelled [*F*_(2,18)_ = 1.584; *p* = 0.2325] as reported in **Figure 6**.

## Discussion

The present study explored the therapeutic potential of Noxiall^®^, a combination of PEA, BCP, myrrh, and carnosic acid, in the treatment of NP elicited by sciatic nerve ligation in the mouse. The rational for the combination of the active ingredients contained in Noxiall^®^ comes from the results of previous studies demonstrating their efficacy in the treatment of chronic pain in various animal model (Dolara et al., 1996; Esposito et al., 2012; Hesselink and Hekker, 2012; Passavanti et al., 2017; Segat et al., 2017). Evidence of clinical efficacy of PEA in NP has been also documented (Hesselink and Hekker, 2012; Gabrielsson et al., 2016).

Consistent with current literature, results showed that sciatic nerve ligation in the mouse led to a significant development of mechanical allodynia that is one of the main symptoms of NP (Kaulaskar et al., 2012; Jensen and Finnerup, 2014). As expected the allodynic response increased progressively after surgery and reached its maximal expression at 4 days after surgery. No signs of spontaneous recovery from allodynia were observed throughout the experimental period (14 days). This is in line with the current literature demonstrating that in CCI rodent models, allodynia remains remarkably stable for at least 1 month from surgery (Bennett and Xie, 1988; Bennett et al., 2003). In our experiment, treatment with Noxiall^®^ elicited a marked enhancement of the withdrawal threshold in response to a mechanical stimulus applied to the ligated paw suggesting an ability of this treatment to attenuate allodynia. In parallel to treatment with Noxiall^®^, a separate group of mice was treated with gabapentin, an anti-epileptic agent that is a first-line treatment for NP in the clinic (Wiffen et al., 2017; Moore et al., 2018). The dose of gabapentin used was chosen based on previous studies in which its efficacy in rodent CCI models was demonstrated (Kusunose et al., 2010; Corona-Ramos et al., 2016; Hewitt et al., 2016). As expected gabapentin was highly effective in attenuating the allodynia associated with sciatic nerve ligation. We found that in the CCI model, Noxiall^®^ was as potent as gabapentin. Based on this finding, we sought, therefore, important to further characterize the pharmacological effects of Noxiall^®^ on thermal hyperalgesia in CCI mice (Espejo and Mir, 1993; Baliki et al., 2005). Results showed that CCI elicited a significant increase in heat sensitivity that was markedly reduced by both Noxiall^®^ and gabapentin. Of note these effects of drugs cannot be attributed to unspecific changes in motor performances as both agents did not significantly modify the distance travelled in the open field test. These observations prompted us to determine the dose response curve of Noxiall^®^ in comparison to pregabalin, another gabapentinoid was widely used for the treatment of NP in the clinic. Also, in this case, the doses of pregabalin were chosen on the base of previous studies in which its efficacy in the treatment of NP was demonstrated in rodents (Kusunose et al., 2010; Corona-Ramos et al., 2016). Results indicated that both treatments significantly attenuated allodynia in a dose related manner. In the case of pregabalin the effect was significant following administration of 25 and 50 mg/kg but not after treatment with 15 mg/kg. Noxiall^®^, also significantly reduced allodynia at all but the lowest dose. Treatments never modified the tactile response at the control paw, suggesting that the effects of Noxiall^®^ and pregabalin were not influenced by potentially unspecific actions such as sedation or loss of mechanical sensitivity (i.e., analgesia). Gabapentinoids represent a first-line treatment in chronic NP and represent one of the most widely used clinical approach to control this condition. Unfortunately, their efficacy appears to be limited as several clinical studies reported that use of these agents is beneficial only in about one out of three or four (Finnerup et al., 2007; Moore et al., 2011; Wiffen et al., 2017). In addition, these compounds, especially when given at high dosages and to elderly patients can produce pronounced side effects, such as excessive sedation, numbness, loss of vigilance, decline in cognitive performances (Moore et al., 2011; Quintero, 2017). On the other hand, low doses of gabapentinoids are often not sufficient to control NP symptoms. prompted by these issues, we decided to determine whether combining the nutraceutical with pregabalin was able to enhance the efficacy of low dose of pregabalin treatment. We treated mice with the low, ineffective dose of pregabalin (15 mg/kg) and the low, ineffective dose of Noxiall^®^. While the low doses of pregabalin and Noxiall^®^ again proved ineffective at attenuating allodynia, interestingly, when the two low doses treatment was combined, we saw a significant attenuation of allodynia. Our finding supports the possibility of using this nutraceutical in association with pregabalin, and possibly with other pharmacological treatment, as an add-on to improve treatment efficacy. The clinical advantage would be better control of NP symptoms at lower pregabalin dosages, and therefore, a lower risk of patients experiencing gabapentinoids side effects.

In the present study, we did not explore the specific role of the individual active ingredients (PEA, BCP, myrrh, and carnosic acid) contained in Noxiall^®^. However, have looked at potential roles for each component in treating NP. PEA has been shown to strongly attenuated neuropathic pain both in rodents and in humans (Costa et al., 2008; Dominguez et al., 2012; Hesselink and Hekker, 2012; Kopsky and Hesselink, 2012; Skaper et al., 2014; Britti et al., 2017). This effect appears to be partly mediated through PPARα activation (LoVerme et al., 2005). Myrrh has also been shown to possess analgesic and anti-inflammatory properties in several animal models of acute pain (Dolara et al., 1996; Shalaby and Hammouda, 2014). The effect of myrrh appears to be mediated by activation of opioid receptors as the analgesic effect of curzarene and furaneudesma-1,3-diene, the two major active compounds of the phytocomplex, is blocked by administration of the opioid antagonist naloxone (Dolara et al., 1996). Beneficial effects of BCP on NP have been demonstrated. Acting as cannabinoid CB2 receptor agonist, BCP has been shown to exerts anti-inflammatory effects and can prevent the recruitment of immune system occurring during development of chronic NP (Howlett et al., 2002; Klauke et al., 2014). Finally, because of its anti-oxidant properties carnosic acid can contribute to attenuate the consequence of neuronal insults occurring in NP condition (Satoh et al., 2008; Ibarra et al., 2011; Maione et al., 2017).

A major finding in our study is the ability of Noxiall^®^ to potentiate the analgesic effects of pregabalin. To show this we used the “Combination Subthresholding” approach which consists in showing that a combination of non-effective doses of two drugs yields significant effects. This method is considered a valid approach to evaluate the enhancement in the activity of two or more biologically active compounds when they are combined (Foucquier and Guedj, 2015). However, the approach is limited in not being able to detect whether the effect is additive or synergistic. For this level of analysis, the “Loewe Additivity” method and isobologram evaluation should be used (Loewe, 1953; Foucquier and Guedj, 2015; Lederer et al., 2018). However, robust isobolograms are difficult to build in *in vivo* studies as they require the test of multiple doses of each single compound and their combinations, making the experiment expensive, technically challenging and prohibitive (Lehar et al., 2007; Zhao et al., 2014). In chronic pain studies, this is further complicated by ethical factors imposing the use of the minimal number of animals possible (Stephens et al., 1998; Flecknell, 2002; Carbone, 2011). Despite this limitation, an enhance efficacy of the combined treatment is evident. The exact mechanism subserving this positive interaction is, at present, unknown. However, we can speculate that gabapentinoids primarily acting on a_2_d_1_-subunit of the voltage-gated calcium (Ca^2+^), reduce neuronal calcium influx at the pre-synaptic nerve (Fink et al., 2002; Bian et al., 2006). This reduction in calcium influx results in an inhibition of the release of neurotransmitters, including norepinephrine (Dooley et al., 2000; Dooley et al., 2002); substance P (Maneuf et al., 2001); and glutamate (Maneuf and McKnight, 2001; Maneuf et al., 2001; Cunningham et al., 2004) and thereby attenuates neuronal excitability associated with sensory transmission. Through its active compounds Noxiall^®^, instead, engages several biological systems as described above exerting anti-inflammatory, immune suppressant and anti-oxidant effects which work to attenuate the chronic stage of NP (Dolara et al., 1996; Esposito et al., 2012; Hesselink and Hekker, 2012; Passavanti et al., 2017; Segat et al., 2017). In conclusion the present study demonstrates that the nutraceutical containing PEA, BCP, myrrh, and carnosic acid and commercially available under the name of Noxiall^©^ reduces mechanically induced allodynia in CCI mice. The effect is dose related and is maintained for the entire treatment period which suggest that it is not subjected to rapid tolerance development. In our CCI model the efficacy of this combination of natural products was comparable to that of gabapentin and pregabalin. Moreover, if co-administered with pregabalin it enhanced the efficacy of the gabapentinoid. Together these findings suggest that the nutraceutical composition contained in Noxiall^®^, may have potential in the treatment of NP and may represent an interesting add-on to treatment with medications used for the management of chronic NP.

## Ethics Statement

All animal manipulations were carried out according to the European Community guidelines for animal care. Formal approval to conduct the experiments described was obtained from the Italian Ministry of Health and the Organism Responsible for Animal Welfare of the University of Camerino (Protocol N 1D580.5).

## Author Contributions

All the authors contributed to design the experiments. Additionally, YF and AE conducted the experiments. YF analyzed the data and wrote the manuscript. AB contributed to prepare the manuscript. RC supervised the study and contributed to the writing of the manuscript.

## Funding

This work was supported by Program EUREKA, of the Region Marche, Italy.

## Conflict of Interest Statement

The PhD fellowship of YF was partially supported by FB-Health. FB-Health had no role in the study design, data collection, analysis, or presentation of the data. RC received an honorarium as member of the Editorial Board of “Focus on Brain,” a scientific journal supported by FB-Health.

The remaining authors declare that the research was conducted in the absence of any commercial or financial relationships that could be construed as a potential conflict of interest. 
