# Supplementary material for: Efficacy of a Combination of N-Palmitoylethanolamide, Beta-Caryophyllene, Carnosic Acid, and Myrrh Extract on Chronic Neuropathic Pain: A Preclinical Study
Source: Front Pharmacol. 2019 Jun 27;10:711. doi: 10.3389/fphar.2019.00711 (PMC6610250; doi:10.3389/fphar.2019.00711)
Supplement: Supplementary file 1 [file Table_1.pdf]

PRODUCT NAME: ROSEMARY EXTRACT CA 65%

CODE: 40322

Ingredients	Rosemary leaf Extract	100 %
Ingredients origin	UE	

Physical – chemicals specifications

Appearance	Yellowish to brownish powder, relatively free flowing, with characteristics flavour and taste**
Solubility	Soluble in alcohol, acetone, and propylene glycol. Insoluble in water. Practically soluble in vegetable oil.

Analytical specifications	Specifications	Method
Loss on drying:	Less than 2%	Eur. Pharm. 8 Met 2.2.32
Heavy metals (as Pb)	Less than 10 ppm	Eur. Pharm. 8 Met 2.4.8. C
Cadmium*	Less than 1 ppm	Eur. Pharm. 8 Met 2.2.58.
Mercury*	Less than 0.1 ppm	Eur. Pharm. 8 Met 2.2.58.
Lead*	Less than 2 ppm	Eur. Pharm. 8 Met 2.2.58.
Arsenic*	Less than 1 ppm	Eur. Pharm. 8 Met 2.2.58.
Carnosic acid (HPLC ,vs standard, as db)	More than 65%	NUTRAFUR MCL04 2004
Residual solvent (Acetone)	Not more than 500 ppm	Eur. Pharm. 8 Met 2.4.24
Aflatoxins B1, B2, G1,G2*	< 4 ppb	HPLC/FLD
Aflatoxins B1*	< 2 ppb	HPLC/FLD
Benzo(a)pyrene*	<10ppb	Regulation EC 333/2007
Sum of Benzo(a)Pyrene, Benzo(a)anthracene, Benzo(b)fluoranthene and chrysene*	<50ppb	Regulation EC 333/2007
Microbiology assay		
Total Plate count	Less than 1000 cfu/g	Eur. Pharm. 8 Met 2.6.12
Yeast and Mould	Less than 100 cfu/g	Eur. Pharm. 8 Met 2.6.12
E. Coli	Negative/g	Eur. Pharm. 8 Met 2.6.13
Salmonella spp	Negative/25g	Eur. Pharm. 8 Met 2.6.13

(*) Analyzed two batches per year, in accordance with Nutrafur HACCP.

(**) Color variations may occur from batch to batch

NUTRAFUR S.A.- P. O. Box. 182, 30820. Alcantarilla (Murcia) SPAIN. PHONE: 00 34 968 892855
FAX: 00 34 968 806512 e-mail: nutrafur@nutrafur.com www.nutrafur.com

URS is a member of Registrar of Standards (Holdings) Ltd.

Pesticides statement In compliance with the correspondent European Regulation for this product.

Absence of foreign matters Over 1 mm (screen).

Nutritional data

Water (%)	0.5-2.0
Carbohydrates (%)	0.5-1.0
Lipids (%)	20.0-25.0
Proteins (Nx 6.25) (%)	0.5-1.0
Total Diterpenes (%)	68.0-75.0
Other Polyphenols	0.5-2.0
Mineral Salts (%)	0.1-0.5
Energetic Value (Kcal/100g)	130-140

Shelflife 12 months unopened stored in a cool and dry location.

Packaging Double food grade propylene bag in cardboard drums.

Storage and distribution Keep well closed and in well ventilated places.

Applications It is used in lipid based Food. Potential regulation of physiological oxidative status.

Status No GMO Product.

Not Irradiated Product.

This product is not at risk concerning BSE/TSE.

Non ETO

HALAL and KOSHER certificate

Legal status: Rosemary extracts are a 100% natural product approved by:

- FDA Revision of 21 Code of Federal Regulation 21 CFR, Part 182-20.
- FEMA NUMBER 2992.
- Regulation (CE) No. 1831/2003
- Regulation 231/2012/EC (E392)

NUTRAFUR S.A.- P. O. Box. 182, 30820. Alcantarilla (Murcia) SPAIN. PHONE: 00 34 968 892855
FAX: 00 34 968 806512 e-mail: nutrafur@nutrafur.com www.nutrafur.com

URS is a member of Registrar of Standards (Holdings) Ltd.

Allergen statement

	ABSENT	PRESENT	CROSS
Cereal containing, gluten and products thereof	X	☐	☐
Crustaceans and products thereof	X	☐	☐
Eggs and products thereof	X	☐	☐
Fish and product thereof	X	☐	☐
Peanuts and products thereof	X	☐	☐
Soybeans and products thereof	X	☐	☐
Milk and products thereof	X	☐	☐
Nuts, namely: almonds (Amygdalus communis L.), hazelnuts (Corylus avellana), walnuts (Juglans regia), cashews (Anacardium occidentale), pecan nuts (Carya illinoinensis (Wangenh.) K. Koch), Brazil nuts (Bertholletia excelsa), pistachio nuts (Pistacia vera), macadamia or Queensland nuts (Macadamia ternifolia), and products thereof, except for nuts used for making alcoholic distillates including ethyl alcohol of agricultural origin	X	☐	☐
Celery and products thereof	X	☐	☐
Mustard and products thereof	X	☐	☐
Sesame seeds and products thereof	X	☐	☐
Sulphur dioxide and sulphites	X	☐	☐
Lupin and products thereof	X	☐	☐
Molluscs and products thereof	X	☐	☐

NUTRAFUR S.A.- P. O. Box. 182, 30820. Alcantarilla (Murcia) SPAIN. PHONE: 00 34 968 892855
FAX: 00 34 968 806512 e-mail: nutrafur@nutrafur.com www.nutrafur.com

UNITED REGISTRAR OF SYSTEMS URS ISO 9001 UKAS MANAGEMENT SYSTEMS 043
URS is a member of Registrar of Standards (Holdings) Ltd.
