# Supplementary material for: Efficacy of a Combination of N-Palmitoylethanolamide, Beta-Caryophyllene, Carnosic Acid, and Myrrh Extract on Chronic Neuropathic Pain: A Preclinical Study
Source: Front Pharmacol. 2019 Jun 27;10:711. doi: 10.3389/fphar.2019.00711 (PMC6610250; doi:10.3389/fphar.2019.00711)
Supplement: Supplementary file 2 [file Table_2.pdf]

biosfered
SPIN-OFF ACCADEMICO
DELL'UNIVERSITÀ DEGLI STUDI DI TORINO

Via G. Quarello 15/A
10135 Torino (TO) Italia
Tel: 011 670 6359
FAX:011 236 6359
www.biosfered.com

Codice prodotto: PNF01	PipeNig®
**Pipenig® -FL**	
Estratto fluido di *Piper nigrum* ad elevato contenuto di β-cariofillene	

Data di Emissione:	Aprile 2017		
Nome botanico e origine:	*Piper nigrum L.* ; India	Numero CAS:	84929-41-9
Parte utilizzata	*fructus, oleum*	EINECS:	284-524-7
Sito di produzione:	Italia	Codice NC:	1302197000

**SPECIFICHE DEL PRODOTTO**	
Solvente d'estrazione:	Acqua

**PRINCIPI ATTIVI**	**Parametro**	**Metodo di analisi**
β-cariofillene (CAS: 87-44-5; EINECS: 201-746-1)	≥ 800 g/Kg (80%)	GC-MS/FID
α-cariofillene (α-umulene) (CAS: 6756-98-6; EINECS: 229-816-7)	≥ 20 g/Kg (2%)	GC-MS/FID
Altri terpeni<br>α-Pinene (CAS: 80-56-8; EINECS: 201-291-9)<br>β-Pinene (CAS: 127-91-3; EINECS: 204-872-5)<br>D,L-Limonene (CAS: 5989-27-5; EINECS: 227-813-5)<br>α-copaene (CAS: 3856-25-5; EINECS: 223-364-4)	≤ 50 g/Kg (5%)	GC-MS/FID

**SPECIFICHE GENERALI**	**Parametro**	**Metodo di analisi**
Aspetto	Liquido giallo paglierino	Ispezione
Ceneri	< 1%	600 °C - 6 ore
Residuo solventi (Reg. 32/2009)		
Etanolo	Assente	
Metanolo	Assente	
Metalli pesanti		
Pb	< 3 ppm	Assorbimento atomico
Cd	< 1 ppm	Assorbimento atomico
Hg	< 0,1 ppm	Assorbimento atomico
As	< 1 ppm	Assorbimento atomico
Contaminazioni microbiche		
TAMC	≤ 5 x 10^4 cfu/g	come per Eur.Ph
TYMC	< 100 cfu/g	come per Eur.Ph
Batteri gram-negativi resistenti ai sali biliari	< 100 cfu/g	come per Eur.Ph
Escherichia coli (1 g)	Assente	come per Eur.Ph
Salmonella (25 g)	Assente	come per Eur.Ph
Cross Contamination	Assente	

Sede amministrativa:
Via Paolo Veronese 202 - 10148 Torino, Italia
Tel 011 348 1511 FAX 011 348 1525
P. IVA:IT10910930014

info@biosfered.com
PEC:biosfered@mypec.eu

SPIN-OFF ACCADEMICO
DELL'UNIVERSITÀ DEGLI STUDI DI TORINO

Via G. Quarello 15/A
10135 Torino (TO) Italia
Tel: 011 670 6359
FAX:011 236 6359
www.biosfered.com

Eccipienti		
Olio di riso (*Oryza sativa* L.	2-15%	Ponderale
Sostanze ausiliari	Assente	
Conservanti	Assente	

**SPECIFICHE GENERALI**	**Parametro**	**Metodo di analisi**
Aflatossine		
Aflatossina B1	< 5 ppb	MI 118/13 rev 1 del 2013
Aflatossine totali (somma di B1, B2, G1 e G2)	< 10 ppb	MI_118/13 rev 1 del 2013
Glutine	≤ 20 ppm	E.L.I.S.A.
Residuo di pesticidi	In accordo con Reg. (EC) No 396/2005 e successive modifiche	GC-MS

**ALTRE INFORMAZIONI**	
Trattamenti conservativi	Il prodotto non è né irradiato né sottoposto a trattamento con ossido di etilene
BSE/TSE/ Vegetariani/Vegani	Il prodotto non contiene materiale di origine animale o implica direttamente e volontariamente l'uccisione, la detenzione o lo sfruttamento di animali.
Nanomateriali	Il prodotto non contiene e non è stato fabbricato utilizzando nanomateriali (Reg.CE N° 1169/2011)
Autocontrollo	Prodotto sottoposto al regime previsto dal programma aziendale di autocontrollo come da pacchetto di igiene Reg. (CE) 852/2004
OGM	Il prodotto non contiene e non è stato fabbricato utilizzando organismi geneticamente modificati (EEC Reg. 1829/2003 e 1830/2003)
Conservazione	Conservare in luogo fresco, asciutto e ventilato, al riparo dalla luce, nei contenitori originali, ben chiusi e lontano da fonti di calore. Sigillare la confezione dopo l'uso.
Interazioni con farmaci	Non sono disponibili informazioni che permettano di stabilire le precauzioni di carattere generale o precauzioni specifiche concernenti interazioni con farmaci, gli effetti teratogeni o non teratogeni in gravidanza, l'allattamento o l'uso pediatrico. Per ulteriori informazioni consultare anche la monografia su Pipenig® prodotta da Biosfered S.r.l. (www.biosfered.com). Questa nota non costituisce parere medico, che va comunque consultato all'insorgenza di un qualsiasi sintomo.
Controindicazioni e avvertenze	Ipersensibilità accertata verso il prodotto
Finalità fisiologiche e salutistiche	Consultare la monografia su Pipenig® prodotta da Biosfered S.r.l. (www.biosfered.com)
Confezionamento	Bottiglie idonee al contatto o al confezionamento di prodotti alimentari.
Data di retest	2 anni dalla data di produzione

SPIN-OFF ACCADEMICO
DELL'UNIVERSITÀ DEGLI STUDI DI TORINO

Via G. Quarello 15/A
10135 Torino (TO) Italia
Tel: 011 670 6359
FAX:011 236 6359
www.biosfered.com

Allergene (Reg. 1169/2011)	ESISTE NEL PRODOTTO?	ESISTE NELLA LINEA PRODUTTIVA?	ESISTE NEL LUOGO DI STOCCAGGIO?
Biossido di zolfo e solfiti (specificare la quantità se più alta di 10 ppm o 10 mg/kg)	NO	NO	NO
Cereali contenti glutine	NO	NO	NO
Di semi di arachidi e prodotti derivati (compreso l'olio)	NO	NO	NO
Anacardio - guscio (compreso l'olio)	NO	NO	NO
Noce (compreso l'olio)	NO	NO	NO
Nocciola (compreso l'olio)	NO	NO	NO
Noce brasiliana (compreso l'olio)	NO	NO	NO
Noce Pecan (compreso l'olio)	NO	NO	NO
Noce Macadamia (compreso l'olio)	NO	NO	NO
Pistacchio - noce (compreso l'olio)	NO	NO	NO
Pinoli (compreso l'olio)	NO	NO	NO
Mandorla (compreso l'olio)	NO	NO	NO
Uova e prodotti derivati	NO	NO	NO
Mollusco e prodotti derivati	NO	NO	NO
Pesci-crostacei e prodotti derivati (inclusa gelatina)	NO	NO	NO
Soia e prodotti derivati (incluso lecitine)	NO	NO	NO
Latte e prodotti lattiero-caseari contenenti lattosio (In caso di presenza specificare la quantità)	NO	NO	NO
Semi di sesamo e prodotti derivati (compreso l'olio)	NO	NO	NO
Lupini (lat.*Lupinus*) e prodotti derivati	NO	NO	NO
Sedano e prodotti derivati	NO	NO	NO
Senape e prodotti derivati	NO	NO	NO
Lattice	NO	NO	NO
Gli eventuali metodi d'analisi non riportati sono metodi interni del produttore ottenibili su specifica richiesta. Le informazioni sopra riportate non Vi sollevano dall'obbligo di identificare il prodotto prima dell'impiego. La nostra società non si assume alcuna responsabilità per danni a persone o cose derivanti dall'impiego dei prodotti da noi commercializzati. Questo documento è proprietà esclusiva di Biosfered S.r.l. ed ogni utilizzo non autorizzato sarà perseguito ai fini di legge. Le informazioni contenute in questo documento sono da intendersi riservate al solo ambito professionale e quindi non divulgabili al pubblico.			
