# Supplementary material for: Efficacy of a Combination of N-Palmitoylethanolamide, Beta-Caryophyllene, Carnosic Acid, and Myrrh Extract on Chronic Neuropathic Pain: A Preclinical Study
Source: Front Pharmacol. 2019 Jun 27;10:711. doi: 10.3389/fphar.2019.00711 (PMC6610250; doi:10.3389/fphar.2019.00711)
Supplement: Supplementary file 4 [file Table_4.pdf]

FRAUPHARMA

TECHNICAL DATA SHEET	Last Version: April 2017

PRODUCT NAME: **PALMITOYLETHANOLAMIDE FINE FLAKES**

CODE PRODUCT: PEAS01
COUNTRY OF ORIGIN: ITALY
PACKAGING: 5, 10 or 25 Kg in paper drum-sealed aluminum-PE-bags

PHYSICOCHEMICAL CHARACTERISTICS	SPECIFICATIONS	METHOD
APPEARANCE	LAMELLA SHAPED , FLAKES	POSITIVE
IDENTIFICATION	POSITIVE (NMR)	POSITIVE
PURITY (%)	≥ 99.0	INT. METHOD
ASSAY (%)	≥ 99.0	INT. METHOD
PALMITOYLDIETHANOLAMIDE (PDEA)	ABSENT	INT. METHOD
TOTAL OTHER ETANOLAMIDES (%)	NMT 1.0	INT. METHOD
TOTAL IMPURITIES (%)	NMT 1.0	INT. METHOD
WATER CONTENT (%)	NMT 1.0	EP; 2.5.12
MELTING POINT (°C)	98.0 – 100.0	EP; 2.5.15
HEAVY METALS (ppm)	NMT 20	INT. METHOD
RESIDUAL SOLVENTS (ppm)	NMT 250	INT. METHOD
BULK DENSITY (G/ML)	0.37 – 0.41	INT. METHOD

MICROBIOLOGICAL	SPECIFICATIONS	METHOD
TOTAL PLATE COUNT (CFU/g)	NMT 1000	EP; 2.6.12
YEAST AND MOLDS (CFU/g)	NMT 100	EP; 2.6.12
ENTEROBATTERIACEE (CFU/g)	NMT 100	EP; 2.6.13
E. COLI (25G)	ABSENT	EP; 2.6.13
SALMONELLA (25G)	ABSENT	EP; 2.6.13

STORAGE

- Store containers at room temperature in a dry place, away from light and direct air exposure.
- Shelf life: 24 months in the original container

Certifications: Palmitoylethanolamide is Kosher certified and suitable for food application

FrauPharma
Via Cascina Trivulzina 13, Agrate Brianza (MB), 20864, Italy
Tel. + 39 039 6898772 Fax +39 039 6890612
